# Treatment of Diphtheria

**Published:** 1881-07

**Authors:** 


Treatment of Diphtheria.—Prof. Oertel says that diphtheria is an infectious disease, caused by fungus designated as "micrococcus diphtheriæ," which, localized in the mouth and pharynx, produces inflammation and fibrinous exudation of the mucous membrane, after an indeterminable length of time. The general infection is kept up by the local. The treatment must be local, and for this there are two indications: 1. The annihilation of the producer of the disease. 2. The removal from the affected parts of the products of disease. For the first, he uses a spray of five per cent. carbolic acid in the throat, often even to the extent of producing the olive green urine. The sprayings are used every two hours, for three to five minutes at a session. If the carbolic acid produces disturbances of digestion, or kidneys, change to benzoate of soda, or boracic acid.

2. To hasten separation of diptheritic membranes there are two methods: (*a*) Suppuration and (*b*) mechanical dislodgement, by exciting mucous secretion. (*a*) Suppuration is hastened by the carbolic sprays, and by inhaling steam impregnated with benzoic acid. The limitation of the membrane is much more speedily induced by these agents. (*b*.) The secretion of the mucous membrane is especially stimulated by jaborandi. The secretion beneath the false membrane increasing raises the false membrane.

Microscopic examination of sections of mucous membrane, covered with thick diphtherial deposits in process of separation, shows that the mucus exuding out of the dilated glandular ducts tends upward in diverse streamlets and breaks through, in part, fibrinous coagula immediately over the ducts, and permeates the network and lifts up the pseudo membrane over a more or less large extent.

Grave constitutional disturbances arise in this disease that must be met by peculiar means, and require comprehensive attention. But it should never be forgotten that the general affection is secondary to the local. A non-recognition or reversal of this fact on the part of the physician has been fatal to many a patient.—*Archives of Laryngology.*

